# Supplementary material for: One N-glycan regulates natural killer cell antibody-dependent cell-mediated cytotoxicity and modulates Fc γ receptor IIIa / CD16a structure
Source: bioRxiv. 2024 Aug 25:2024.06.17.599285. Originally published 2024 Jun 18. Preprint. [Version 2] doi: 10.1101/2024.06.17.599285 (PMC11212880; doi:10.1101/2024.06.17.599285)
Supplement: 2 [file NIHPP2024.06.17.599285v2-supplement-2.pdf]

bioRxiv preprint doi: https://doi.org/10.1101/2024.06.17.599285; this version posted August 25, 2024. The copyright holder for this preprint (which was not certified by peer review) is the author/funder, who has granted bioRxiv a license to display the preprint in perpetuity. It is made available under aCC-BY 4.0 International license.

Supplemental Figures and Tables for

One N-glycan regulates natural killer cell antibody-dependent cell-mediated cytotoxicity and modulates Fc γ receptor IIIa / CD16a structure

Paul G. Kremer[1], Elizabeth A. Lampros[1], Allison M. Blocker[1], Adam W. Barb[1,2,3*]

[1]Department of Biochemistry and Molecular Biology, University of Georgia, Athens, GA

[2]Complex Carbohydrate Research Center, University of Georgia, Athens, GA

[3]Department of Chemistry, University of Georgia, Athens, GA

bioRxiv preprint doi: https://doi.org/10.1101/2024.06.17.599285; this version posted August 25, 2024. The copyright holder for this preprint (which was not certified by peer review) is the author/funder, who has granted bioRxiv a license to display the preprint in perpetuity. It is made available under aCC-BY 4.0 International license.

Supplemental Table 1. Binding affinity measurements for each FcγRIIIa variant

	Binding to fucosylated IgG1 Fc		Binding to afucosylated IgG1 Fc	
FcγR3a Variant	K_D (nM)	StdDev (nM)	K_D (nM)	StdDev (nM)
EndoF F153S	35.07	22.98	44.40	38.18
EndoF R155S	105.77	13.44	86.27	3.57
EndoF S164A	111.73	54.12	118.80	41.30
EndoF S164A F153S	243.00	59.40	167.00	55.15
EndoF S164A V158F	381.50	62.93	320.00	72.12
EndoF S164A R155S	116.00	18.38	124.50	16.26
EndoF T167Y	261.00	151.32	225.50	95.46
EndoF V158	21.74	5.41	17.88	6.61
EndoF Y132S	12876.67	7035.73	7530.00	1547.87
F153S	322.75	144.04	133.95	44.51
V158F	944.75	228.50	238.75	58.96
G129D	>10000	n.a.	>10000	n.a.
H119A	477.00	100.80	225.67	78.78
H134A	771.67	251.14	339.67	80.03
I88A	360.33	269.47	227.77	239.49
K120A	1385.00	529.79	597.00	142.28
K128A	659.67	68.97	316.67	175.30
K128A R155S	n.b.	n.a.	n.b.	n.a.
K131A	549.00	223.76	330.60	211.95
K161A	2723.33	1201.68	1153.33	55.08
L124A	734.67	134.94	339.00	198.55
L157S	324.33	44.41	475.67	304.08
N162Q	200.00	103.58	118.30	44.83
R155S	637.67	117.59	619.00	435.44
S160A	326.00	142.04	131.53	59.71
S164A	159.13	76.79	92.19	67.71
S164A F153S	136.33	5.86	93.13	20.82
S164A V158F	1105.00	35.36	710.50	154.86
S164A I158	124.55	64.28	87.70	38.61
S164A K128A	252.50	58.69	189.00	84.85
S164A Man 3	108.53	42.75	15.43	3.88
S164A Man1	95.93	31.82	n.t.	n.a.
S164A N	131.98	34.70	n.t.	n.a.
S164A NG	112.45	50.15	n.t.	n.a.
S164A NGG	106.35	49.22	n.t.	n.a.
S164A R155S	128.30	88.67	94.25	71.77
S164A T171A	147.35	140.93	132.70	110.73
T122A	1197.67	462.08	640.33	221.41
T167Y	229.00	62.22	164.00	58.56
T171A	n.b.	n.a.	555.66	1031.54
V158	305.35	130.44	168.37	295.84
V158 Man 1	95.00	53.53	n.t.	n.a.
V158 Man3	27.50	14.65	15.63	3.42
V158 N	123.15	33.57	20.90	12.54
V158 NG	109.92	22.05	17.57	9.95
V158 NGG	87.78	24.99	13.71	7.19
V158I	210.27	98.28	101.77	67.45
W113A	n.b.	n.a.	>10000	n.a.
W90A	n.b.	n.a.	9040.00	1559.52
Y132S	n.b.	n.a.	>10000	n.a.

n.b.- no binding detected
n.a. - not applicable
n.t. - not tested

bioRxiv preprint doi: https://doi.org/10.1101/2024.06.17.599285; this version posted August 25, 2024. The copyright holder for this preprint (which was not certified by peer review) is the author/funder, who has granted bioRxiv a license to display the preprint in perpetuity. It is made available under aCC-BY 4.0 International license.

Supplemental Table 2. Raw ADCC data from the lentivirus transduced YTS cells.*

date	V158 RTX	V158 aRTX	V158 +K RTX	V158 +K aRTX	V158F RTX	V158F aRTX	V158F +K RTX	V158F +K aRTX	S164A RTX	S164A aRTX	S164A +K RTX	S164A +K aRTX	T167Y RTX	T167Y aRTX	T167Y +K RTX	T167Y +K aRTX
	9.2	14.8	9.4	14.2	0.9	11.7	1	16.1	10.5	8.4	6.2	-2	9.7	36.4	12.1	39.3
12/6/23	9.7	13.8	11.6	15.2	1.9	11.3	1.3	11.1	9.5	4.4	6.6	4.9	5.4	46.7	15.8	54.8
	7	13.5	9.6	26.2	-0.6	22.7	2.4	30.9	7.7	8.3	9.7	13.8	7.3	66.5	20.5	63.8
	3.3	35.2	25.2	42.9	1.9	18.6	3	25.4	21.2	25.1	17.8	25.1	9.4	9	10.5	40
12/13/23	17.7	40.1	23.5	39.5	1.4	15.1	4.3	28.7	19.1	23.9	19.7	23.2	8.3	17.6	20.6	26.7
	17.7	38.6	24.4	43.1	-0.2	18.1	3.6	21.6	14.7	24.3	19.6	21.7	9.6	20.3	20.8	38.3
	14.6	45.5	24.4	51.8	12.4	18.2	16.8	24.7	0.6	19.1	4.6	30.8	19.4	52.1	26.7	39
12/20/23	13.8	47.5	30.1	54.2	10.6	31	19.5	28.1	2.9	34.4	4.8	36.2	24.1	53.1	24.6	36.84
	14.7	39	25.8	44.6	12.4	29.2	13.6	22.1	0.3	31	3.6	37.9	14.5	42.7	21.4	40.44
average	12	32	20	37	5	20	7	23	9.6	20	10.3	21	12	38	19	42

*V158= wt; aRTX = afucosylated rituximab, K=Kifunensine treatment

bioRxiv preprint doi: https://doi.org/10.1101/2024.06.17.599285; this version posted August 25, 2024. The copyright holder for this preprint (which was not certified by peer review) is the author/funder, who has granted bioRxiv a license to display the preprint in perpetuity. It is made available under aCC-BY 4.0 International license.

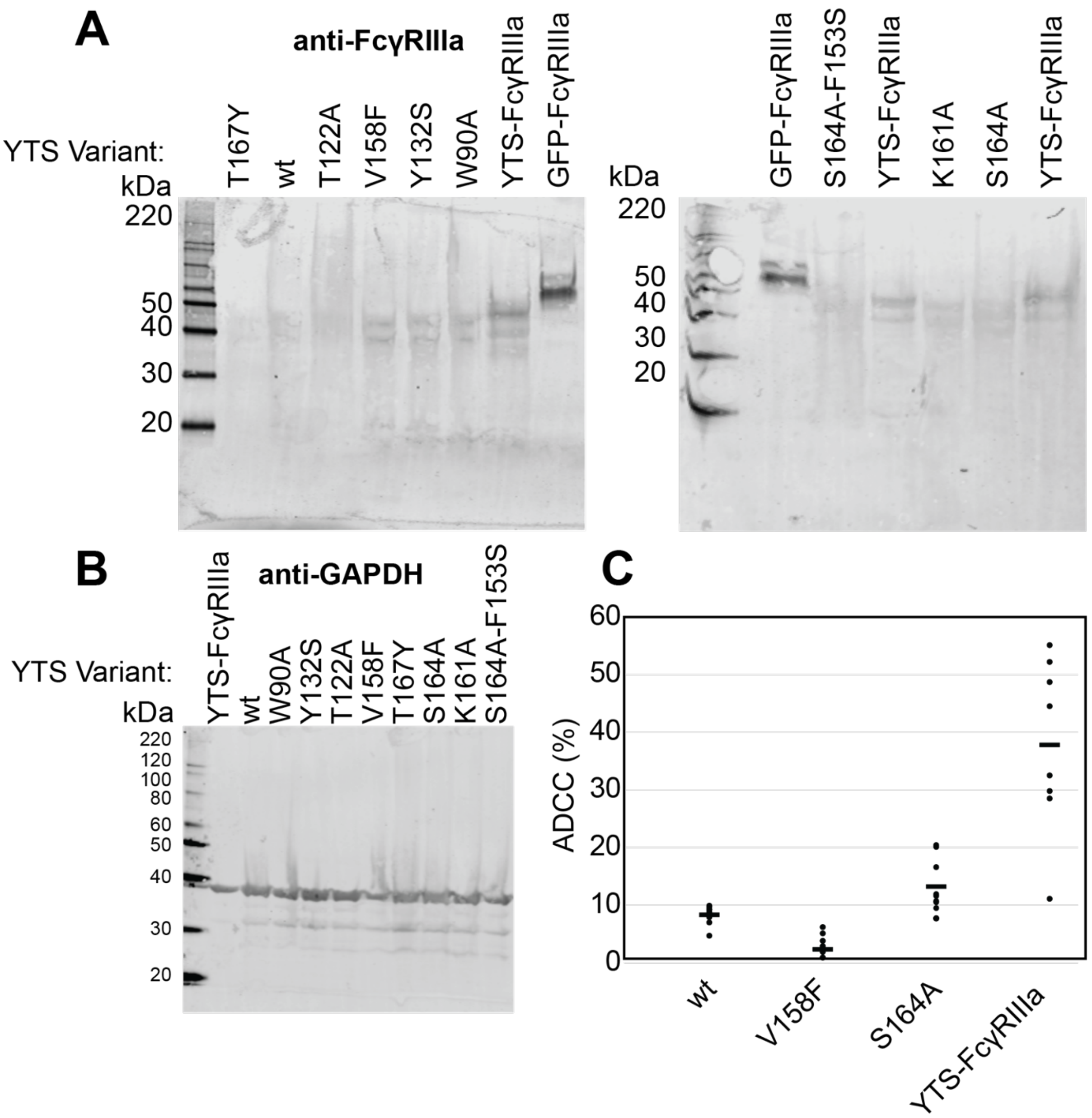

Supplemental Figure 1. Comparison of expression and ADCC of the previously-established YTS-FcγRIIIa NK cell line and the lentivirus-transduced cell lines prepared herein. **A.** Western blots showing expression levels compared to 40 ng of recombinant GFP-FcγRIIIa protein. "YTS-FcγRIIIa" refers to the previously established YTS-FcγRIIIa" cell line in contrast to the lenti-virus transduced YTS cells expressing various FcγRIIIa variants described herein. **B**. GAPDH expression levels of the YTS cells. **C.** The ADCC of the YTS-FcγRIIIa cell line is greater than our YTS cells transduced with FcγRIIIa V158. Data shown include three independent experiments collected on different days, each with three replicates.

bioRxiv preprint doi: https://doi.org/10.1101/2024.06.17.599285; this version posted August 25, 2024. The copyright holder for this preprint (which was not certified by peer review) is the author/funder, who has granted bioRxiv a license to display the preprint in perpetuity. It is made available under aCC-BY 4.0 International license.

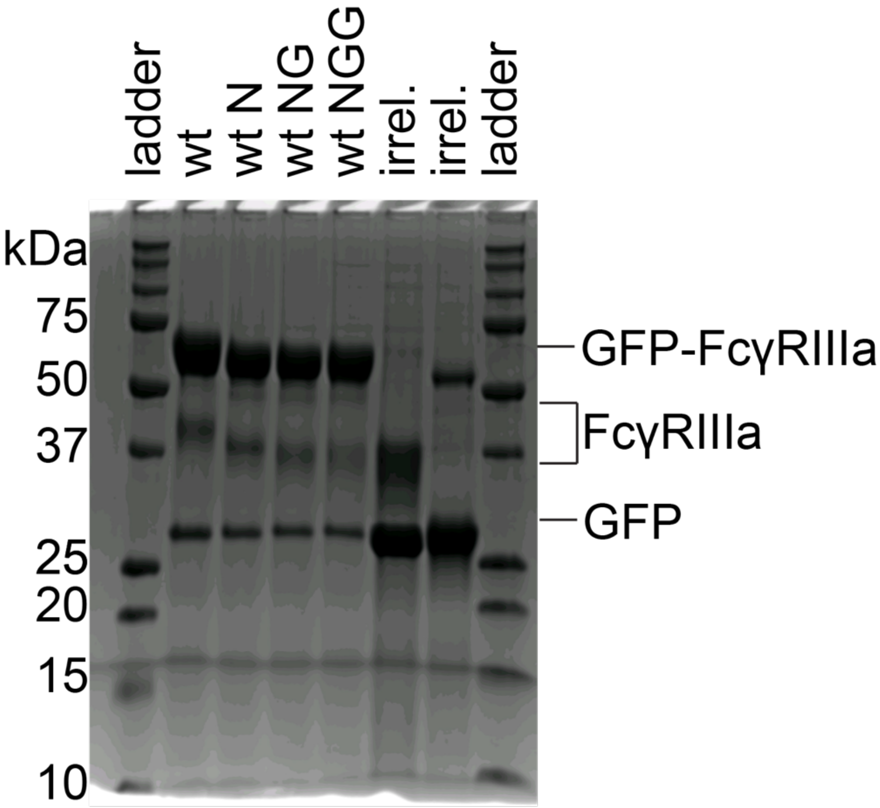

Supplemental Figure 2. Glycosidase digestions visualized by SDS-PAGE. GFP-FcγRIIIa was treated with various glycosidases, revealing a stepwise increase in migration rate on a reducing SDS-PAGE gel. N= neuraminidase treated. NG=treatment with neuraminidase and galactosidase. NGG=treatement with neuraminidase, galactosidase and N-acetylglucosaminidase. Analyses of endoglycosidase F products are characterized elsewhere (Lampros et al. (2022) Curr Res Immunol 3:128-135).

bioRxiv preprint doi: https://doi.org/10.1101/2024.06.17.599285; this version posted August 25, 2024. The copyright holder for this preprint (which was not certified by peer review) is the author/funder, who has granted bioRxiv a license to display the preprint in perpetuity. It is made available under aCC-BY 4.0 International license.

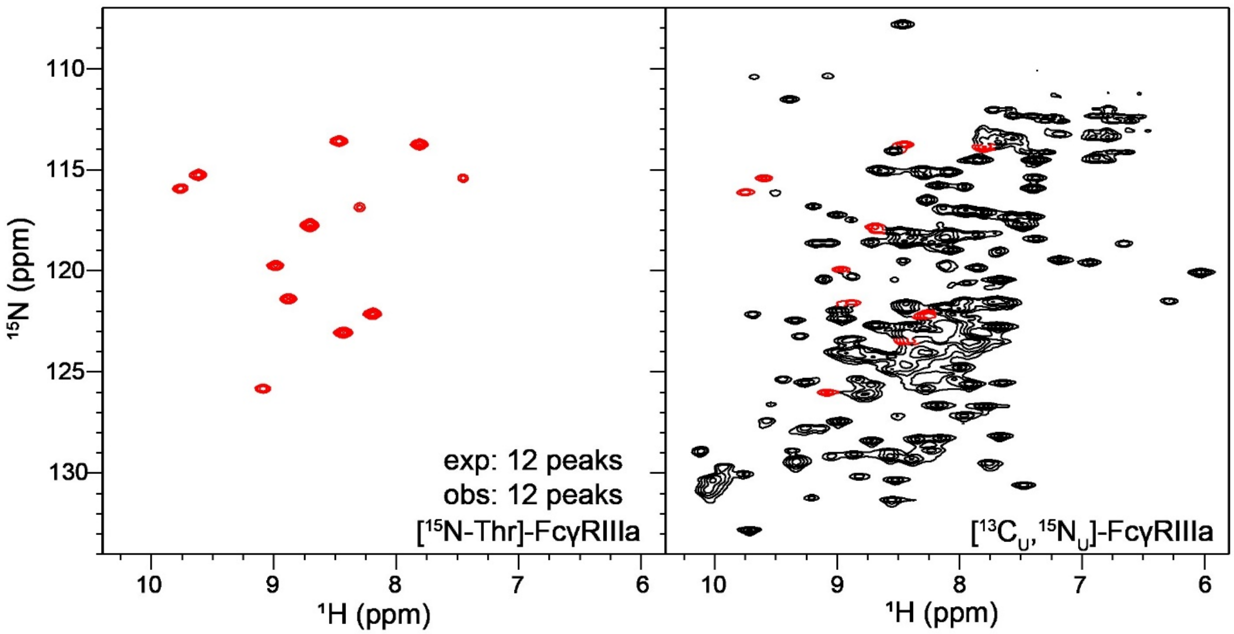

Supplemental Figure 3. Residue-specific labeling of the glycosylated FcγRIIIa. A. FcγRIIIa with two N-glycans expressed from HEK293F cells grown in medium supplemented with [^{15}N]-Threonine. **B.** Peaks in the spectrum of [^{13}C,^{15}N]-FcγRIIIa corresponding to threonine residues (*red* contours).

bioRxiv preprint doi: https://doi.org/10.1101/2024.06.17.599285; this version posted August 25, 2024. The copyright holder for this preprint (which was not certified by peer review) is the author/funder, who has granted bioRxiv a license to display the preprint in perpetuity. It is made available under aCC-BY 4.0 International license.

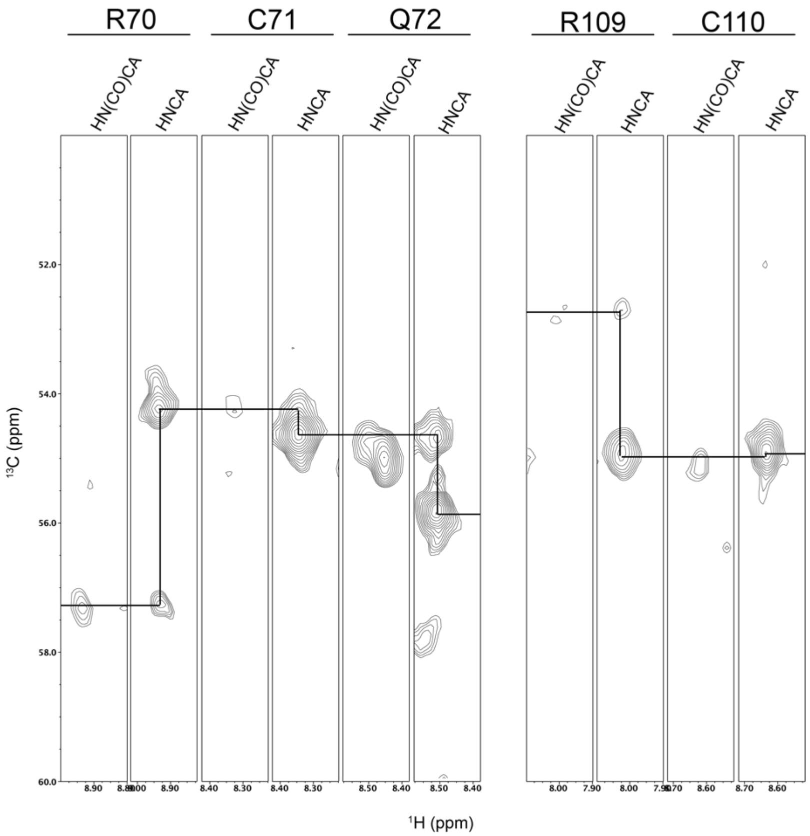

Supplemental Figure 4. Strip plots from triple resonance HNCA and HN(CO)CA experiments showing the assignments of the C71 and C110 resonances. The resonances for H111 were not identified. Residue connectivities are shown with a black line.

bioRxiv preprint doi: https://doi.org/10.1101/2024.06.17.599285; this version posted August 25, 2024. The copyright holder for this preprint (which was not certified by peer review) is the author/funder, who has granted bioRxiv a license to display the preprint in perpetuity. It is made available under aCC-BY 4.0 International license.

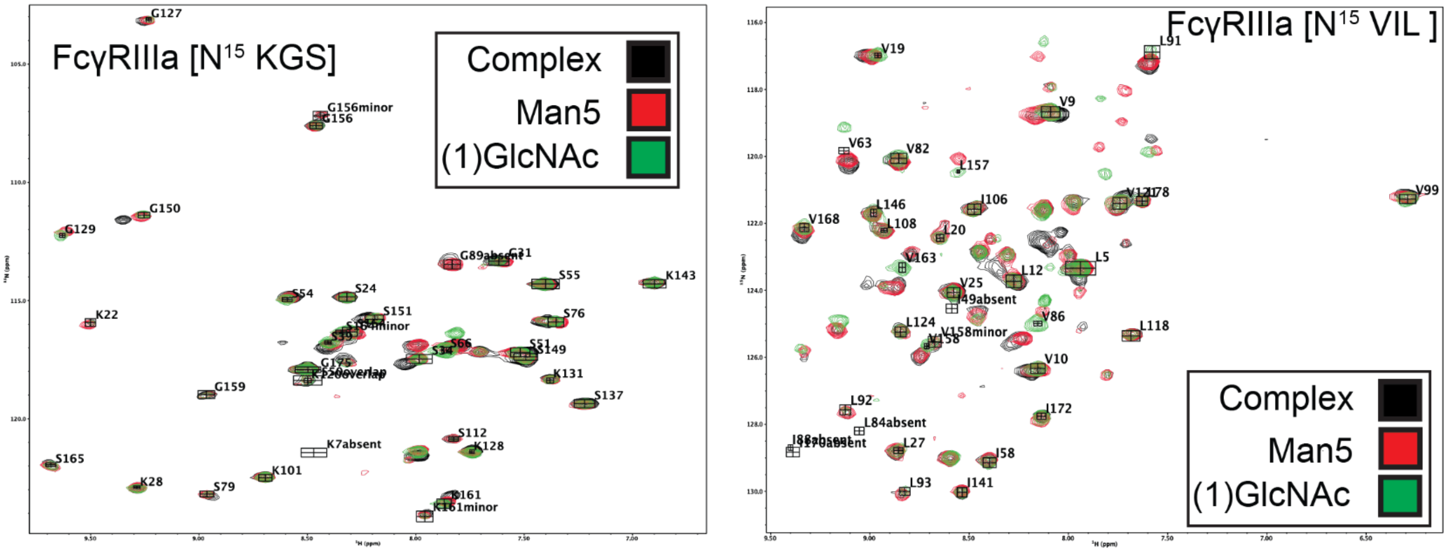

Supplemental Figure 5. Overlayed HSQC-TROSY spectra of ^{15}N-labeled FcγRIIIa with complex-type (*black*) 5-mannose (*red*) or (1)GlcNAc (*green*) N-glycans.

bioRxiv preprint doi: https://doi.org/10.1101/2024.06.17.599285; this version posted August 25, 2024. The copyright holder for this preprint (which was not certified by peer review) is the author/funder, who has granted bioRxiv a license to display the preprint in perpetuity. It is made available under aCC-BY 4.0 International license.

A

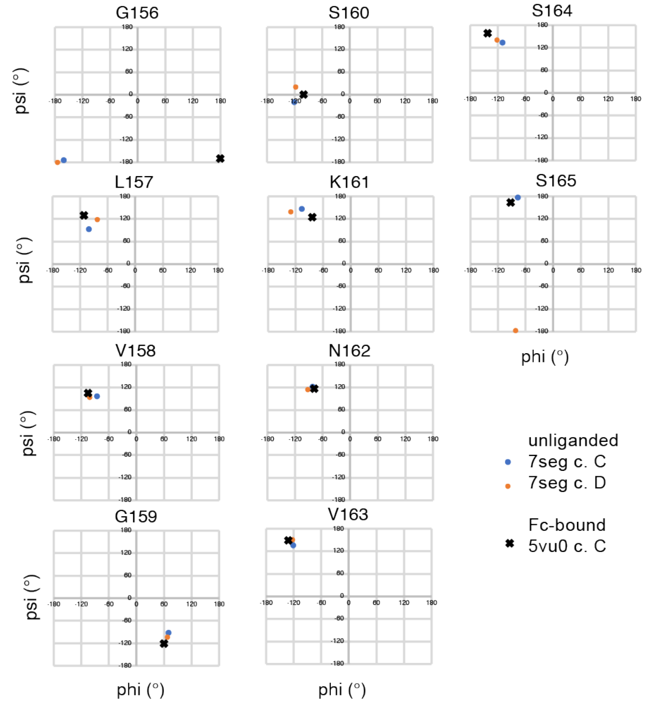

B

	7seg c. C		7seg c. D		5vu0 c. C	
Residue	phi (°)	psi (°)	phi (°)	psi (°)	phi (°)	psi (°)
153	-164	157	-147	166	-153	167
154	-119	157	-128	167	-126	164
155	-144	136	-160	150	-162	160
156	-161	-174	-174	-180	180	-170
157	-101	93	-83	118	-112	130
158	-86	97	-102	94	-105	106
159	70	-92	67	-103	60	-121
160	-121	-19	-118	20	-101	1
161	-107	147	-131	139	-84	124
162	-82	122	-92	115	-78	117
163	-121	136	-122	151	-132	149
164	-110	134	-122	141	-142	159
165	-77	177	-82	-178	-92	164
166	-78	174	-88	157	-74	166
167	-91	140	-82	152	-83	157
168	-127	122	-126	132	-139	147
169	-89	112	-89	108	-102	131
170	-103	128	-102	126	-116	125

Supplemental Figure 6. Backbone dihedral angles from two FcγRIIIa X-ray crystallography structures. **A.** Phi-Psi angle plots for select residues in the FG loop. **B.** Table of Phi-Psi angles for select FcγRIIIa residues. 7seg and 5vu0 are pdb IDs for the two datasets analyzed.
